# Relation entre les caractéristiques des table-bancs et les mesures anthropométriques des écoliers au Benin

**DOI:** 10.11604/pamj.2014.17.284.3719

**Published:** 2014-04-15

**Authors:** Stève Marjelin Falola, Polycarpe Gouthon, Jean-Marie Falola, Michel Armand Fiogbe, Issiako Bio Nigan

**Affiliations:** 1 Laboratoire de Biomécanique et Performance, Institut National de la Jeunesse, de l'Education Physique et du Sport (INJEPS), Université d'Abomey-Calavi, Bénin; 2 Laboratoire APS et Motricité, Institut National de la Jeunesse, de l'Education Physique et du Sport (INJEPS), Université d'Abomey-Calavi, Bénin; 3 Laboratoire Motricité Humaine, Education, Sport, Santé (LMHESS), Faculté des Sciences du Sport, Université de Nice Sophia Antipolis, France; 4 Unité de Recherche en Chirurgie Pédiatrique (URCP), Faculté des Sciences de la Santé, Université d'Abomey-Calavi, Bénin

**Keywords:** Mobilier scolaire, école primaire, enfant, anthropométrie, République du Bénin, school furniture, primary school, child, anthropometry, Republic of Benin

## Abstract

**Introduction:**

Le mobilier scolaire et la posture assise en classe sont souvent impliqués dans l'apparition des douleurs rachidiennes, influant de fait sur la qualité des tâches réalisées par les apprenants. Aucune étude n'a encore vérifié le degré d'adéquation entre les caractéristiques du mobilier et celles des écoliers au Bénin. L'objectif de cette étude transversale est donc de déterminer la relation entre les dimensions des table-bancs utilisées en classe et les mesures anthropométriques des écoliers au Bénin.

**Methods:**

Elle a été réalisée avec un échantillon probabiliste de 678 écoliers, âgés de 4 à 17 ans. Les mesures anthropométriques des écoliers et les mensurations relatives aux longueurs, largeurs et hauteurs des table-bancs ont été mesurées, puis intégrées aux équations proposées dans la littérature. Les pourcentages des valeurs situées hors des limitesacceptables, dérivées de l'application des équations ont été calculés.

**Results:**

La largeur et la hauteur des table-bancs utilisées par les écoliers étaient plus élevées (p < 0,05) que les valeurs de référence recommandées par les structures officielles de contrôle et de production des mobiliers scolaires au Bénin. Quel que soit le sexe, il y avait une inadéquation entre la largeur du banc et la longueur fesse-poplité, puis entre la hauteur de la table et la distance coude-bancdes écoliers.

**Conclusion:**

Les résultats suggèrent de prendre en compte l’évolution des mesures anthropométriques des écoliers dans la confection des table-bancs, afin de promouvoir de bonnes postures assises en classe et de réduire le risque de troubles du rachis.

## Introduction

L’école, deuxième maison de l'enfant est l'environnement au sein duquel il passe environ cinq heures par jour en position assise pour accomplir ses tâches scolaires [1]. Il passe environ 80% du temps scolaire en classe, exerçant diverses activités telles que la lecture, l’écriture, le dessin et d'autres activités connexes qui l'obligent à s'asseoir de façon continue pendant des heures [2]. La posture assise est par excellence la position de travail à l’école [3]. Elle est également une posture éprouvante pour le rachis car elle modifie ses courbures physiologiques [4]. En posture assise, le bas du dos est en effet soumis à une contrainte bien plus importante que lorsque l'on est debout ou couché. La posture assise provoque des contraintes correspondant à 140% du poids du corps au niveau des disques intervertébraux [5]. Chez les enfants scolarisés, ses conséquences sont déterminées par les activités menées en classe, les mesures anthropométriques, les dimensions et le modèle de mobilier scolaire utilisé [6]. Les écoliers sont plus exposés aux affections provoquées par la position assise prolongée sur un mobilier inadapté [7]. Un mobilier inadapté à la morphologie de l'enfant et un cartable trop lourd ont été reconnus commefacteurs partiellement impliqués dans la rachialgie, dans de nombreux cas [8]. Les mesures anthropométriques des écoliers doivent être par conséquent prises en compte lors de la confection du mobilier scolaire, afin de garantir et de promouvoir de bonnes postures assises des écoliers en classe [9]. Les effets nocifs (rachialgies, troubles musculosquelettiques) d'un mobilier scolaire inadapté sur le rachis et les apprentissages scolaires des écoliers ont été mis en évidence [2–10]. Le degré d'adéquation entre les dimensions du mobilier scolaire et les caractéristiques morphologiques des écoliers a été également évalué dans différents pays. En Iran et en Corée par exemple,du fait des différences d’âges et de sexes, des inadéquationsentre les dimensions du mobilier scolaire et les caractéristiques morphologiques des écoliers ont été rapportées [11, 12]. Aux USA, seuls 18,9% des tables et des chaises étaient adaptés aux écoliers américains âgés de 11 à 13 ans [7]. Chez des enfants grecques de 7 à 12 ans,la longueur du siège n’était adaptée à aucun écolier du second degréet la hauteur du siège n’était adaptéequ’à 5% des enfants [13]. En Indonésie, les écoliers souffrent de douleurs sur plusieurs segments osseux, surtout au cou (61,3%), aux épaules (57%), à la taille (49,2%), aux fesses (54,5%) et aux bras (72,3%)du fait de l'inadéquation entre les mesures anthropométriques des écoliers et les dimensions du mobilier scolaire [14].

En Afrique, précisément au Nigéria, une différence significative entre les mesures anthropométriques des enfants de 5 à 14 ans et les dimensions des mobiliers scolaires utilisés a été observée [15]. Si une telle démarche d’évaluation a permis de constater dans ces pays, que le mobilier scolaire n’était pas adapté aux caractéristiques anthropométriques des écoliers, aucune étude allant dans ce sens n'a été réalisée au Bénin. Par ailleurs, lestable-bancs utilisés par l’écolier du Bénin ne fait pas non plus objet de modification normative à mesure que ce dernier grandit et passe d'une classe à l'autre [16]. La présente étude a par conséquent pour objectif de déterminer la relation entre les dimensions des table-bancsutilisés par les enfants inscrits à l’école primaire au Béninet leurs caractéristiques morphologiques.

## Méthodes

**Nature de l’étude:** Il s'agit d'une étude transversale descriptive et analytique, entrepriseau Bénin, d'avril à fin juin 2012. Elle a reçu l'approbation du Comité Scientifique Sectoriel des Sciences et Techniques des Activités Physiques et Sportives (CSS/STAPS) de l'Université d'Abomey-Calavi qui tenait lieu de Comité d’éthique.

**Population d’étude et échantillonnage:** L’étude a été réalisée avec les garçons et les filles inscrits dans les classes de deuxième année des cours préparatoire, élémentaire et moyen (CP, CE2, CM2) des écoles primaires publiques (EPP). L’échantillon d’étude a été constitué par la méthode probabiliste et la technique de sondage à six degrés(communes, circonscriptions scolaires, écoles, groupes pédagogiques, classes et écoliers) dans les 12 départements administratifs du Bénin,retenus de façon systématique. Les communes ont été choisies dans chaque département,par la technique aléatoire simpleà partir de la liste officielle [17]et dans les communes, l’échantillonnage en grappe à deux degrés a été utilisépourretenir les 15 écoles correspondant aux circonscriptions scolaires ciblées. Le choix des écoles, groupes pédagogiques et table-bancs dans les classes a été effectué par tirage au sort dans lesécoles. Les classes de CP, CE2 et CM2 représentant les trois cycles du primaire, ont été retenues de façon systématique par groupe pédagogique, de sorte queles écoliers avec leurs mobiliers respectifsont été choisis en utilisant le registre des enseignants dans 45 classes. La taille de l’échantillon d’étude,déterminée à partir de la formule de Schwartz [18], d'un taux d'absence estimé à 10% et sur la base de 15 élèves par classe était de 678 écoliers, dont 360 filles et 318 garçons. Les critères d'inclusion dans l’échantillon d’étude se présentent comme suit: être inscrit ou inscrite dans l'une des classes de CP, CE2 ou CM2 des écoles retenues, être sain (e) d'apparence, c'est-à-dire ne souffrir d'aucune pathologie rachidienne, comme en témoigne le livret de santé scolaire actualisé de l'année en cours. Les écoliers handicapés des membres inférieurs n'ont pas été intégrés à l’échantillon d’étude. Les parents ou les tuteurs des écoliers, ainsi que les Directeurs des écoles ciblées ont donné leur consentement éclairé écrit pour la participation des enfants à l’étude.

**Matériels et techniques:** Les mesures de la masse corporelle, de la taille debout et assise ont été effectuées en utilisant respectivement un pèse personne à affichage digitalBR 9012 (Camry, Chine), précis à 0,1 kg près et une toise murale 206 M (Seca-Bodymeter, France) graduée au millimètre près. Les dimensions du mobilier scolaire et les longueurs segmentaires des écoliers ont été mesurées à l'aide d'un mètre ruban Butterfly en toile non extensible et d'une règle graduée conçue à cet effet. Lesparamètres anthropométriques et les dimensions du mobilier scolaire ont été mesurés selon la technique recommandée [19-20], les écoliers ayant adopté la posture assise habituelle en classe.

**Collecte des données:** Une pré-enquête auprès des structures publiques chargées du contrôle et de la production du mobilier scolaire a permis de disposer des documents relatifs aux normesofficielles de fabrication des table-bancs au Bénin [16–21]. Pour chaque paramètre anthropométrique, la moyenne de trois mesures successives a été retenue. Les autres données ont été collectées une seule foischez tous les écoliers, en français, en langues nationales fon, mina, yoruba ou dendi,selon leur groupe ethnolinguistique d'origineet dans les mêmes conditions.

**Variables étudiées:** L’âge, le sexe et le type de posture assise adoptée avec deux modalités (posture assise face au tableau PAFT et posture assise de profil par rapport au tableau PAPT), ainsi que les dimensions des table-bancs ont été déterminés. Les paramètres anthropométriques étaient l'indice de masse corporelle (IMC), la taille debout, la taille assise,le tour de taille,les hauteursdu poplité, coude-bancet genou-sol,les longueursdu brasetfesse-poplité, la distance bitrochantérienne. Les dimensions des table-bancs et les mesures anthropométriques ont été intégréesdans cinqparmi les relations combinatoires proposéespar Gouvali et Boudolos [22], pour déterminer chez chaque écolier, l'existence ou non d'adéquation entre ses mesures anthropométriques et les dimensions de sa table-banc. Il s'agissait de:

1. (Hauteur du poplité + 2) cos 30° ≤ ***Hauteur du banc*** ≤ (Hauteur du poplité + 2) cos 5°
2. (0,80 x Longueur fesse-poplité) ≤ ***Largeur du banc*** ≤ (0,99 x Longueur fesse-poplité)
3. (1,1 x Distance bitrochantérienne) ≤ ***Longueur du banc*** ≤ (1,30 x Distance bitrochantérienne)
4. Hauteur coude-banc + (Hauteur du poplité + 2) cos 30° ≤ ***Hauteur de la table*** ≤ (Hauteur du poplité + 2) cos 5° + (Hauteur coude-banc x 0,8517) + (Longueur du bras x 0,1483)
5. (Hauteur genou-sol +2) + 2 ≤ ***Hauteur dessous-table-sol*** ≤ (Hauteur du poplité + 2) cos 5° + (Hauteur coude-banc x 0,852) + (Longueur du bras x 0,148) - 4

Dans chaque cas, la relation a été considérée comme adéquate lorsque les valeurs obtenues en intégrant les mesures, se situaient dans les intervalles tels que définis. Toutes les valeursqui s’étaient retrouvées hors desintervalles, ont été considérées comme traduisant une inadéquation entre la caractéristique du mobilier en question et les paramètres anthropométriques de l’écolier.

**Analyse statistique:** Les données ont été traitées en utilisant le logiciel Statistica de Stat Soft Inc. (version 6. 0). Pour chaque variable, des statistiques descriptives sous forme de moyenne ± écart type ont été calculées. Les fréquences relatives des valeurs situées au-delà et en deçà des valeurs limites (minimales et maximales)acceptables dans chaque relationont été déterminées. Le niveau de signification des tests statistiques a été fixé à p < 0,05.

## Résultats

**Caractéristiques des écoliers:** l’échantillon d’étude était composé de 360 filles (53,1%) et de 318 garçons (46,9%) âgés de 4 à 17 ans (Tableau 1). Les filles étaient âgées en moyenne de 9,6 ± 2,3 ans, pour une taille et un poids moyensrespectifs de 133,0 ± 14,9 cm et 29,0 ± 9,7 kg, contre9,8 ± 2,6 ans, 132,8 ± 15,2 cmet 28,8 ± 9,9 kg chez les garçons (p > 0,05). Aucun des écoliers n'avait un IMC > 30 kg/m^2^. Les filles avaient toutefois un périmètre abdominal en moyenne plus élevé que celui des garçons (p < 0,05).

**Tableau 1:** Caractéristiques biométriques des écoliers

	Filles (n = 360)	Garçons (n = 318)	Echantillon total (n = 678)
Age (ans)	9,6 ± 2,3	9,8 ± 2,6	9,7 ± 2,5
MC (kg)	29,0 ± 9,7	28,8 ± 9,9	28,9 ± 9,8
Taille debout (cm)	133,0 ± 14,9	132,8 ± 15,2	132,9 ± 15,0
Taille assise (cm)	65,6 ± 7,5	65,3 ± 6,6	65,5 ± 7,1
IMC (kg/m^2^)	15,9 ± 2,2	15,8 ± 2,2	15,9 ± 2,2
TH (cm)	64,5 ± 9,1	63,1 ± 8,9*	63,8 ± 9,0

Les nombres dans les cases représentent les valeurs moyennes ± écarts types; MC: masse corporelle; IMC: indice de masse corporelle; TH: tour de hanche; n: effectif

* Différence entre garçons et filles, significative à p < 0,05.

**Dimensions du mobilier scolaire:** la longueur des bancsétait en moyenneplus élevée chez les filles (p = 0,002) que chez les garçons(Tableau 2). Les tablesutilisées par les écoliers avaient dans 66,6% des cas, une hauteur supérieure à la valeur recommandéepar les structures officielles chargées du contrôle et de la production des mobiliers scolaires au Bénin (Tableau 3). Chez47,3% des écoliers, lalongueur des tables était inférieure à la valeur recommandée. La longueurdes bancs était inférieure à la valeur recommandéechez 64,7% de l’échantillon et la hauteur plus grandechez 59,4% (Tableau 4).

**Tableau 2:** Dimensions du mobilier scolaire utilisé par les écoliers au Bénin

	Filles (n = 360)	Garçons (n = 318)	Echantillon total (n = 678)
**TABLE**			
Longueur (cm)	120,0 ± 2,8	120,0 ± 3,0	120,0 ± 2,9
Largeur (cm)	33,2 ± 3,9	33,7 ± 3,9	33,4 ± 3,9
Hauteur interne (cm)	56,8 ± 4,5	57,0 ± 6,1	56,9 ± 5,3
Hauteur avant (cm)	72,7 ± 3,4	73,0 ± 2,1	72,9 ± 2,9
**BANC**			
Longueur (cm)	120,0 ± 1,4	119,6 ± 1,6*	119,8 ± 1,5
Largeur (cm)	27,0 ± 27,9	24,8 ± 17,2	26,0 ± 23,5
Hauteur (cm)	41,6 ± 1,6	41,7 ± 1,9	41,7 ± 1,7
Distance table-banc I (cm)	19,0 ± 3,7	19,0 ± 3,8	19,0 ± 3,7
Distance table-banc S (cm)	22,0 ± 4,5	22,3 ± 4,9	22,2 ± 4,7

Les nombres dans les cases représentent les valeurs moyennes ± écarts types;Hauteur interne (table): distance entre le sol et le bord inférieur du casier; Hauteur avant (table): distance entre le sol et le bord avant du casier;Distance table-banc I: distance entre la table et le banc à la partie inférieure du mobilier; Distance table-banc S: distance entre la table et le banc à la partie supérieure du mobilier; n: effectif

* Différence entre garçons et filles, significative à p < 0,05.

**Tableau 3:** Comparaison entre les dimensions du mobilier scolaire utilisé par les écoliers avec les recommandations de la DIEM

	DIEM	Mobiliers des écoliers
**TABLE**		
Longueur (cm)	120,0	120,0
Largeur (cm)	38,0	33,4*
Hauteur (cm)	72,0	72,9*
**BANC**		
Longueur (cm)	120,0	119,8*
Largeur (cm)	24,5	26,0*
Hauteur (cm)	40,0	41,7*

DIEM: Direction des infrastructures, de l’équipement et du matériel du Ministère de l'Enseignement Maternel et Primaire du Bénin

* Différence significative à p < 0,05.

**Tableau 4:** Adéquation entre les dimensions des table-bancs utilisées par les écoliers et les dimensions recommandées par la DIEM

	Tableau (cm) n = 678			Banc (cm) n = 678		
	Longueur	Largeur	Hauteur	Longueur	Largeur	Hauteur
≥ norme	42 (5,9%)	121 (17,2%)	467 (66,6%)	63 (8,9%)	37 (5,2%)	417 (59,4%)
Norme	304 (43,3%)	508 (72,4%)	118 (16,8%)	161 (22,9%)	612 (87,3%)	26 (3,7%)
< norme	332 (47,3%)	49 (6,9%)	93 (13,2%)	454 (64,7%)	29 (4,1%)	235 (33,5%)

DIEM: DIEM: Direction des infrastructures, de l’équipement et du matériel du Ministère de l'Enseignement Maternel et Primaire du Bénin; ≥ norme: valeur supérieure à celle recommandée par la DIEM (respectivement, 120 cm, 38 cm, 72 cm pour la table et 120 cm, 24,5 cm et 40 cm pour le banc); < norme: valeur inférieure à celle recommandée par la DIEM (respectivement, 120 cm, 38 cm, 72 cm pour la table et 120 cm, 24,5 cm et 40 cm pour le banc); n: nombre de table-bancs associées aux écoliers de l’étude; DIEM: Direction des infrastructures, de l’équipement et du matériel du Ministère de l'Enseignement Maternel et Primaire du Bénin.

**Adéquation entre les mesures anthropométriques et dimensions des table-bancs:** quelque soit le sexe, il y avait une inadéquation entre la largeur du banc et la longueur fesse-poplité des écoliers étudiés, c'est-à-dire quela largeur du banc étaitplus petite que la longueur fesse-poplité chez 99% d'entre eux. La longueur du banc, la hauteur de la table et la hauteur dessous table-sol ont présenté des fréquences d'inadéquation respectivementavec la distance bitrochantérienne chez 95,1% des écoliers, la hauteur coude-banc chez 100% et la hauteur genou-sol chez 97,5% (Tableau 5).

**Tableau 5:** Fréquences relatives des inadéquations des dimensions des table-bancs avec les mesures des écoliers

	Relation 1	Relation 2	Relation 3	Relation 5	Relation 6
	Hauteur du banc	Largeur du banc	Longueur du banc	Hauteur de la table	Dessous table-sol
Valeur ≤ au minimale	192 (28,3%)	671 (99,0%)	6 (0,9%)	-	15 (2,2%)
Valeur minimale admise	486 (71,7%)	7 (1,0%)	672 (99,1%)	678 (100%)	663 (97,8%)
Valeur maximale admise	547 (80,7%)	678 (100%)	33 (4,9%)	-	17 (2,5%)
Valeur ≥ au maximale	131 (19,3%)	-	645 (95,1%)	678 (100%)	661 (97,5%)

Valeur ≤ au minimale: valeur en dessous de la valeur minimale; valeur ≥ au maximale: valeur au dessus de la valeur maximale.

## Discussion

Cette étude a été réalisée avec un échantillon probabiliste constitué en tenant compte à la fois du découpage administratif et territorial, ainsi que de la structure du système scolaire de l'enseignement primaireau Bénin. Les 678 enfants qui y ont participé, peuvent donc être considérés comme représentatifs de l'ensemble des écoliers de ce pays et les résultats présentés, suffisamment fiables pour rendre compte de la réalité dans tout le Bénin. Les mesures ont été en effet effectuées trois fois successivement et la moyenne retenue chez tous les écoliers, par les mêmes évaluateurs expérimentés. Compte tenu de la moyenne d’âge de l’échantillon qui est de 9,7 ans, il est permis de considérer que 63% d'entre eux avaient moins de 12 ans et étaient donc prépubères au moment de l’étude, car le pic de croissance pubertaire est atteint vers l’âge de 12 ans [23]. Malheureusement, cette catégorie d’âge serait laplus exposée aux pathologies rachidiennes associées aux mauvaises postures [23], ce qui renforce davantage la portée de cette étude. Par ailleurs, aucun des écoliers n’était obèse au moment de l’étude. Les douleurs ressenties dans le dos au moins une fois dans le passé par 11% parmi eux,sont probablement associées à d'autres facteurs comme l'hérédité, la station assise prolongée adoptée en classe (> 4 heures) et la disposition adoptée par rapport au tableau [1–24].

La longueur du banc est apparue plus élevée chez les filles que chez les garçons. De plus, des écarts ont été observés entre d'une part les recommandations relatives à la largeur, à la hauteur de la table, à la longueur,à la largeur,la hauteur du banc et d'autre part les mesures enregistrées. Ces résultats mettent en évidence le non-respect des normes de fabrication des table-bancs au primaire par les ébénistes et les fournisseurs de mobilier aux écoles. En effet, malgré les recommandations de la Direction des infrastructures, de l’équipement et du matériel(DIEM) [16], la largeur et la hauteur des table-bancs utilisées par les écolierssont plus élevées que les valeurs de références. Contrairement aux attentes, les dimensions du mobilier scolaire sont les mêmes quels que soient l’âge et le niveau d’étude des écoliers intégrés à cette étude. Cette situation est tout-à-fait anormale, puisque les caractéristiques du mobilier devraient évoluer en fonction de l’âge des écoliers [3–23]. Cela serait bien possible si le mobilier comprenait un dispositif permettant d'en régler les dimensions pour les adapter aux mesures de chaque enfant. Le mobilier scolaire, tel qu'il se présente actuellement, oblige certains écoliers à avancer les fesses au bord des bancs et à se courber pour écrire, puisque leurs pieds ne sont pas en contact avec le sol. Cela pourrait être à l'origine d'un accroissement des douleurs rachidiennes, du fait d'un manque de support pour les cuisses [10–25]. Une inadéquation entre les dimensions des tables-bancs et les mesures anthropométriques a été mise en évidence chez plus d'un écolier sur deux et cela pour presque toutes les dimensions du mobilier.

L'inadéquation observée entre la largeur du banc et la longueur fesse-poplité des écoliers pourrait être due à la petite largeur des bancs qui peut surtout donner à l'enfant, le sentiment qu'il va tomber [25]. La longueur des bancs est également apparue trop grande pour presque tous les écoliers. Il faut noter que la relation numéro 3,relative à la longueur du bancutilisé par les écoliersa été obtenue à partir des mobiliers de type table-chaise individuelle et non table-banc. Dans le contexte béninois où le mobilier utilisé est plutôt une de table-banc, une longueur de banc de 120 cm [16] est considérée comme acceptable. Il a été en effet indiqué que la longueur du banc doit à la fois être suffisante pour soutenir les tubérosités ischiatiques pour garantir la stabilité etlaisser suffisamment d′espace pour faciliter les mouvements latéraux [25, 26]. Si l'on prend le modèle de table-banc utilisé dans l'enseignement primaire au Bénin, la longueur du banc peut être considérée comme assez grande pour permettre à chaque enfant de réaliser ses tâches scolaires sans être gêné par son voisin immédiat. La hauteur de la tableapparaît par contre trop élevée pour les écoliers étudiés, ce qui modifie la posture assise des enfants, en leur imposant une plus grande flexion dans la zone lombaire [23]. Dans ces conditions, l’écolier peut ressentir des douleurs dans tout le dos, lors des tâches d’écriture et de lecture au tableau. Pour réduire ce risque, il faut tenir compte des valeurs minimales et maximales acceptables, telles qu'elles ont été déterminées à partir des cinq relations utilisées dans cette étude [22]. Ces valeurs sont présentées dans le Tableau 6.

**Tableau 6:** Valeurs minimales et maximales acceptables des dimensions pour le mobilier scolaire

	Valeurs minimales acceptables	Valeurs maximales acceptables
**TABLE**		
Hauteur (cm)	47,5	127,2
Hauteur dessous-table-sol (cm)	36	123,2
**BANC**		
Longueur (cm)	26,4	83,2
Largeur (cm)	18,4	69,3
Hauteur (cm)	27,7	74,7

## Conclusion

Cette étude a été mise en oeuvre en posant l'hypothèse que le mobilier utilisé dans l'enseignement primaire au Bénin n'est pas en adéquation avec les caractéristiques anthropométrique des écoliers. La confrontation des données collectées dans les écoles sur le mobilier avec les mesures des écoliers confirment cette hypothèse. Elle révèle même que les normes de confection du mobilier élaborées par le Ministère de l'enseignement Maternel et Primaire (MEMP), Direction des Infrastructures, de l'Equipement et du Matériel (DIEM), sont peu respectées. Ainsi, les table-bancs présentent des mesures soit plus élevées, soit plus basses que celles recommandées,ce qui peut influencer négativement le rendement des écoliers, voire provoquer des dorsalgies. En attendant de disposer de données relatives aux conséquences des mauvaises postures sur l'activité des muscles et les courbures du rachis, il convient que le MEMP mette en place un système de contrôle rigoureux de la qualité du mobilier scolaire. Cela permettra de réduire chez les écoliers, le risque de douleurs rachidiennes liées aux mauvaises postures en classe.
